# Alterated integrin expression in lichen planopilaris

**DOI:** 10.1186/1746-160X-3-11

**Published:** 2007-02-08

**Authors:** Roberto d'Ovidio, Concetta Sgarra, Anna Conserva, Umberto Filippo Angelotti, Roberta Erriquez, Caterina Foti

**Affiliations:** 1 Specialist in Dermatology-AIDA/Tricologia, Bari, Italy; 2 Department of Internal Medicine, Immunology and Infectious Diseases, Unit of Internal Medicine, University of Bari, Policlinico, Piazza Giulio Cesare 11, 70124 Bari, Italy; 3 Department of Internal Medicine, Immunology and Infectious Diseases, Unit of Dermatology, University of Bari, Policlinico, Piazza Giulio Cesare 11, 70124 Bari, Italy

## Abstract

**Background:**

Lichen planopilaris (LPP) is an inflammatory disease characterized by a lymphomononuclear infiltrate surrounding the isthmus and infundibulum of the hair follicle of the scalp, that evolves into atrophic/scarring alopecia. In the active phase of the disease hairs are easily plucked with anagen-like hair-roots. In this study we focused on the expression of integrins and basement membrane components of the hair follicle in active LPP lesions.

**Methods:**

Scalp biopsies were taken in 10 patients with LPP and in 5 normal controls. Using monoclonal antibodies against α_3_β_1 _and α_6_β_4 _integrins we showed the expression of these integrins and of the basement membrane components of the hair follicle in active LPP lesions and in healthy scalp skin.

**Results:**

In the LPP involved areas, α_3_β_1 _was distributed in a pericellular pattern, the α_6 _subunit was present with a basolateral distribution while the β_4 _subunit showed discontinuous expression at the basal pole and occasionally, basolateral staining of the hair follicle. Conclusion: An altered distribution of the integrins in active LPP lesions can explain the phenomenon of easy pulling-out of the hair with a "gelatinous" root-sheath.

## Introduction

Lichen planopilaris (LPP) is an uncommon disease characterized by widespread keratosis pilaris and a progressive inflammatory cicatricial alopecia. The scalp often is the only site of involvement with different manifestations, such as perifollicular erythema and scaling (keratotic spines), patchy or diffuse hair loss, and at the end stage an atrophic cicatricial alopecia with loss of follicular ostia [1-3].

This disease mostly affects middle-aged females without racial predilection. The etiopathogenesis is unknown, although there is an immune infiltrate characterized by actived T lymphocytes (CD4 and CD8), that attacks the epithelium of the infundibula and isthmus areas of hair follicles (the buldge area) and sometimes the interfollicular epidermis with the lower portion of hair follicle being relatively spared [4]. Later findings are perifollicular fibrosis and "artifactual clefts" between epithelium and stroma [5]. Ultimately hair follicles are definively destroyed. The follicular antigen(s) targeted by these lymphocytes are not known. These antigens are thought to be viral, pharmacological or self [4].

In the early stage typical pathohistologic finding of LPP is a lichenoid infiltrates around the follicular epithelium of the infundibuloisthmic region (the bulge area),

Direct immunofluorescence often demonstrates colloid bodies and a linear deposit of fibrin and/or fibrinogen at the dermo-epidermal junction [6].

Tipically in the active phase of the disease there is a positive pull test with hairs that are easily plucked with anagen-like hair-roots presenting dysplastic hair roots sheaths. The outer root sheath cells interact with the basement membrane mainly via α_3_β_1 _and α_6_β_4 _integrins, which are expressed at different levels in different regions of the hair follicle [7].

In particular the aim of this study was to evaluated the expressions of α_3_β_1 _ed α_6_β_4 _integrins of follicular keratinocytes in LPP, comparing that on pathological skin of 10 patients with LPP and 5 healty patients.

## Methods

Patients attending the Unit of Dermatology of the University of Bari with diagnosis of LPP of the scalp based on clinical, histologycal and immunofluorescence findings. The patients were seen from February to November 2005 (2 men and 8 were women) aging range 18–58 years. Clinical and laboratorial data of the 10 patients with LPP are reported in table 1.

**Table 1:** Clinical and laboratorial data of 10 patients with LPP.

**Patients**	**Sex**	**Age**	**Duration of disease**	Granular deposits at DEJ*
1	M	53	10 months	IgM, F
2	F	40	1 year	IgM, F
3	F	58	1 year	Neg
4	M	48	2 years	IgM, F
5	F	18	1 year	Neg
6	F	20	18 months	Neg
7	F	35	3 years	IgM, F
8	F	24	9 months	Neg
9	F	44	2 years	IgM, F, C_3_
10	F	28	7 months	IgM, F

* DEJ – Dermo-epidermal junction

Scalp biopsies were also done in 5 normal scalp controls (2 men and 3 women). The age of the controls range from 19 and 70 years. Informed written consense was obtained from all patients before surgical biopsy. Scalp biopsies consisted of 4 mm punch biopsies taken from the active edges of the lesions and from uninvolved scalp skin in the patients with diagnosis of LPP, and from the mid or posterior crown in the controls. Surgical biopsy specimens were snap-frozen in liquid nitrogen, mounted in Optimal Cutting Temperature 4583 embedding compound (Miles Laboratories, Inc; Naperville, Illinois), and stored at -80°C.

Five-micrometer serial frozen sections were cut with a microtome (Microtom, HM 505E, Carl Zeiss, Oberkochen, Germany), transferred onto glass slides (Sigma Chemical Company) and processed for indirect alkaline phosphatase.

Briefly, sections were fixed in chloroform/acetone mixture for 10 minutes at 4°C, air dried and incubated with primary antibody (10 μg/ml) diluted in RMPI medium containing 10% FCS.

After washing, sections were incubated with secondary antibody (rabbit anti-mouse), washed and incubated with a mouse anti-alkaline phosphatase conjugated antibody (Dako, Glostrup, Denmark). Reactions were developed with red fucsin, sections were counterstained with Mayer's hemalum solution, mounted with glycerol and examined with a Nikon Eclipse photomicroscope (Nikon Corp.).

Antibodies F2, F4, and F1 (to human integrin α_3_) were kindly provided by L. Zardi (IST Genoa, Italy). Antibodies MAR6 and MAR4 (to human integrin α_6 _and β_1 _respectively) were a gift from S. Menard (INT, Milan, Italy). α_6 _(GoH3) was purchased from Pharmingen (San Diego, CA). Monoclonal antibody anti-Ln-1 and Coll IV were purchased from Sigma (St. Louis, MO). Human anti-Ln-5 (GB3) (Vailly et al, 1994) was a gift of G. Meneguzzi (School of Medicine, Nice, France).

## Results

**Figure 1:**
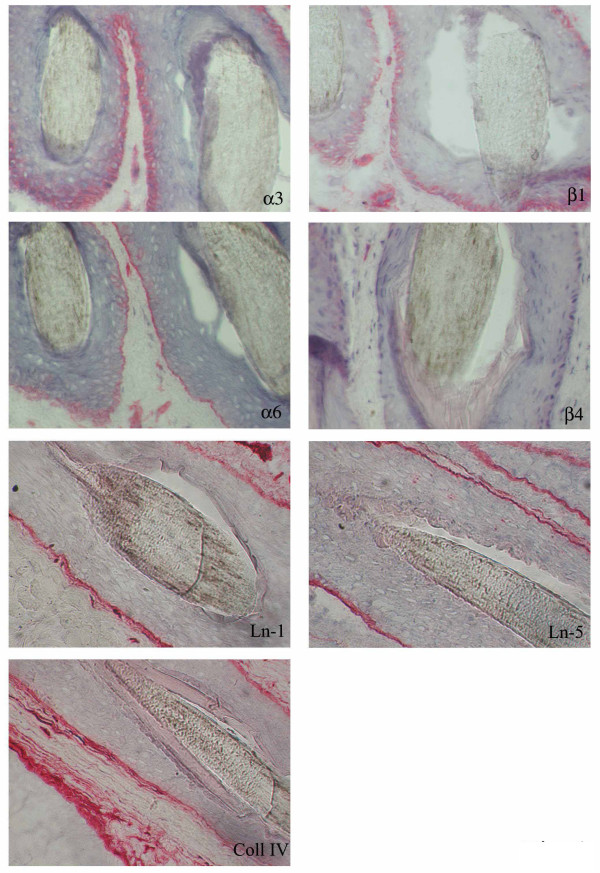
Ln-1, Ln-5, Coll IV, α3β1 and α6β4 integrins staining in a hair follicle from uninvolved scalp skin of a healthy subject.

**Figure 2:**
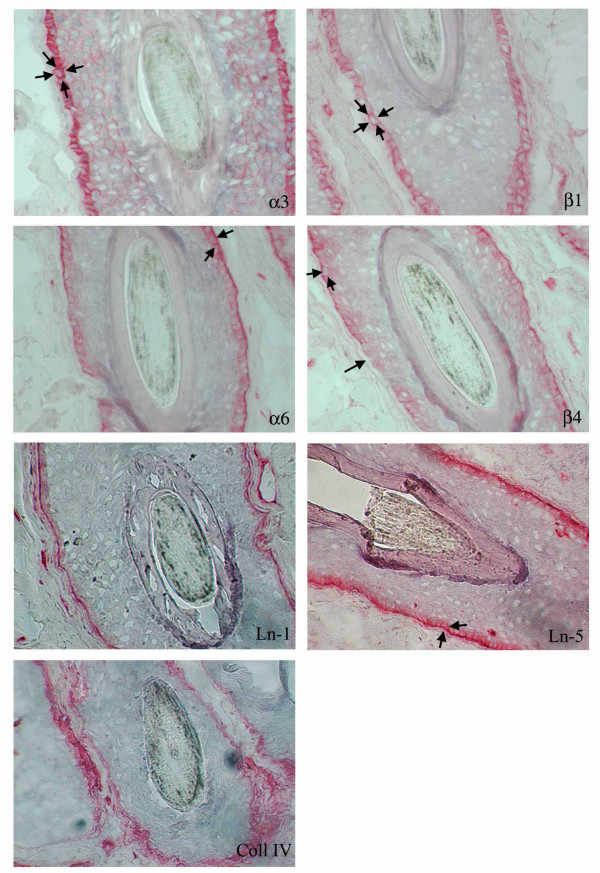
Ln-1, Ln-5, Coll IV, α3β1 and α6β4 integrins staining in a hair follicle from involved scalp skin of a patient with LLP.

In the uninvolved areas integrin α_3_β_1 _was expressed with a basolateral distribution instead α_6_β_4 _was distributed in the basal keratinocyte layer in the Outer Rooth Sheath (ORS) of the follicle (Fig 1). In the involved areas of LPP α_3_β_1 _was distributed in a pericellular pattern, α_6 _subunit was present in a basolateral distribution, while β_4 _subunit shows a discontinuous expression at the basal pole and occasionally basolateral staining of the hair follicle (Fig. 2). Staining for Ln-1 and collagen IV was similar represented in uninvolved (Fig. 1) and involved (Fig. 2) areas of scalp skin. In involved areas of scalp skin Ln-5 (colocalized with α_6 _integrin), showed a basolateral distribution as well as integrin α_6 _(Fig. 2), instead in normal control Ln-5 and integrin α_6 _were distributed along the basal layer of the hair follicle (Fig. 1).

## Discussion

LPP is an inflammatory disease characterized by a lichenoid inflammation surrounding the isthmus and infundibulum of the hair follicle of the scalp and other body areas, that evolves with atrophic cicatricial alopecia.

In this study we focused on the expression of integrins and basement membrane (BM) components of the hair follicle in active LPP lesions and in healthy subjects. Integrin α_6_β_4_, a component of hemidesomsomes, is normally confined to the basal keratinocytes of the hair follicle and has functions involving keratinocyte recognition and attachment to the BM. Integrin α_3_β_1_, a receptor for laminin and collagen, is localized on the basolateral layer of the basal keratinocytes, suggesting that it also has a role in cell-cell adhesion.

In a previous study (performed in mice), Brakebusch et al. demonstrated that β_1 _integrins have an important role in hair follicle morphogenesis, in the differentiation and in the cell-cell interaction of follicular keratinocytes. On the other hand, altered β_1 _integrin expression in follicular keratinocytes provokes dermal fibrosis and hair loss [8].

This study showed an altered distribution of integrins in follicular keratinocytes in LPP involved areas. Integrin α_3_β_1 _was distributed in a pericellular pattern, the α_6 _subunit was present with a basolateral distribution while the β_4 _subunit showed discontinuous expression at the basal pole, and occasionally basolaterally. In uninvolved skin of the scalp the distribution of α_6_β_4 _was exclusively basal. These pathological aspects are also found in erosive lichen planus vulvae and cutaneous lichen ruber planus [9,10].

## Conclusion

This study showed an altered distribution of integrins in follicular keratinocytes in LPP involved areas. The altered expression of integrins may be provoked by cytokines and proteases released by peri- and intra-follicular T lymphocytes, mast cells and macrophages [11,12].

**Figure 3:**
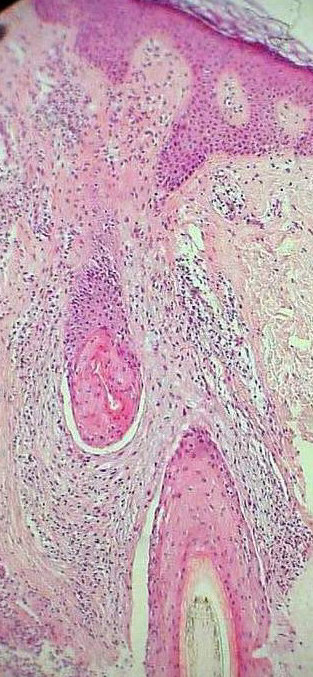
An artifactual cleft between the epithelium and the stroma of the scalp skin in a patient with LPP. Original magnification 100×.

**Figure 4:**
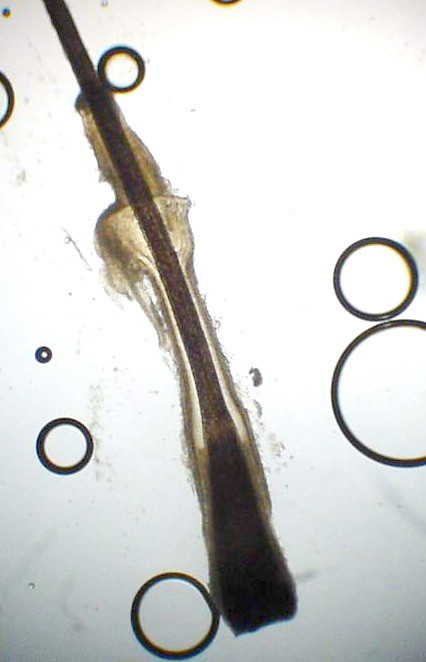
Pulled-out dysplastic anagen hair: the ORS contains remnants of the sebaceous gland at the level of the isthmus.

Altered expression of integrins provokes the loss of adhesion of follicular keratinocytes to the stroma and could explain the artifactual clefts observed in histological samples (Fig. 3) and the phenomenon of easy plucking of the hair, with a "gelatinous" root-sheath containing stem cells (Fig. 4).

For this reason, scratching due to pruritus, but also during normal treatment such ascombing and shampooing, as well as inadequate topical therapy(e.g. ointment), could accelerate the process of irreversible defluvium.

## Competing interests

The author(s) declare they have no competing interests.

## Authors' contributions

DR conceived the study and did the case record search, coordinated the write-up and submission of the article. FC and CA were responsible to see the patients at hospital. AUF carried out the literature search. SC and ER performed the immunohistochemical staining. FC revised the manuscript.
